# Effectiveness of hierarchical medical system and economic growth: based on China’s urban vs. rural health perspectives

**DOI:** 10.3389/fpubh.2024.1364584

**Published:** 2024-05-10

**Authors:** Yongze Zhao, Qingyu Qiao, Xian Xu, Ying Bian

**Affiliations:** ^1^Department of Public Health and Medicinal Administration, Faculty of Health Sciences, University of Macau, Macau, China; ^2^Department of Accounting and Information Management, Faculty of Business Administration, University of Macau, Macau, China; ^3^School of Clinical Medicine, Kangda College of Nanjing Medical University, Lianyungang, China; ^4^Institute of Chinese Medical Sciences, University of Macau, Macau, China; ^5^State Key Laboratory of Quality Research in Chinese Medicine, University of Macau, Macau, China

**Keywords:** hierarchical medical system, urban rural disparities, factor analysis, vector autoregression, China

## Abstract

**Background:**

The hierarchical medical system is an important measure to promote equitable healthcare and sustain economic development. As the population’s consumption level rises, the demand for healthcare services also increases. Based on urban and rural perspectives in China, this study aims to investigate the effectiveness of the hierarchical medical system and its relationship with economic development in China.

**Materials and methods:**

The study analyses panel data collected from Chinese government authorities, covering the period from 2009 to 2022. According to China’s regional development policy, China is divided into the following regions: Eastern, Middle, Western, and Northeastern. Urban and rural component factors were downscaled using principal component analysis (PCA). The factor score formula combined with Urban–rural disparity rate (ΔD) were utilized to construct models for evaluating the effectiveness of the hierarchical medical system from an urban–rural perspective. A Vector Autoregression model is then constructed to analyze the dynamic relationship between the effects of the hierarchical medical system and economic growth, and to predict potential future changes.

**Results:**

Three principal factors were extracted. The contributions of the three principal factors were 38.132, 27.662, and 23.028%. In 2021, the hierarchical medical systems worked well in Henan (*F* = 47245.887), Shandong (*F* = 45999.640), and Guangdong (*F* = 42856.163). The Northeast (ΔD_max_ = 18.77%) and Eastern region (ΔD_max_ = 26.04%) had smaller disparities than the Middle (ΔD_max_ = 49.25%) and Western region (ΔD_max_ = 56.70%). Vector autoregression model reveals a long-term cointegration relationship between economic development and the healthcare burden for both urban and rural residents (β_urban_ = 3.09, β_rural_ = 3.66), as well as the number of individuals receiving health education (β = −0.3492). Both the Granger causality test and impulse response analysis validate the existence of a substantial time lag between the impact of the hierarchical medical system and economic growth.

**Conclusion:**

Residents in urban areas are more affected by economic factors, while those in rural areas are more influenced by time considerations. The urban rural disparity in the hierarchical medical system is associated with the level of economic development of the region. When formulating policies for economically relevant hierarchical medical systems, it is important to consider the impact of longer lags.

## 1 Introduction

**Figure 1:**
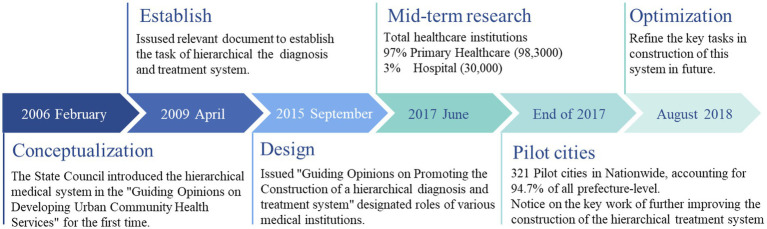
The progress of Chinese hierarchical diagnosis and treatment system.

The hierarchical healthcare system is a service model that seeks to rationalize the allocation of medical resources and optimize the distribution of medical services based on the ranking of medical institutions, the complexity of medical services, and disease severity. The graduated approach to diagnosis and treatment strives to enhance the methodical allocation and utilization of medical resources. This will be achieved through the establishment of a hierarchy of medical institutions based on the severity of the patient’s illness, as determined by the medical institutions. This vision provides a clear division of labor and rationalization of healthcare. Eventually, it can sustain the healthcare sector’s development and ensure that the public can access healthcare while safeguarding their welfare. In China, the past 10 years has been the time when Chinese government promote the urban and rural residents basic medical insurance by integrating the urban residents’ basic medical insurance and new rural cooperative medical scheme (1). As shown in Figure 1, the construction of China’s hierarchical medical system is gradually being developed and improved. Since the implementation of the healthcare reform in 2009, China’s hierarchical medical system has undergone a gradual process of development and enhancement in policy (2). Key tactics applied consist of primary care, two-way referrals, separation of acute and chronic conditions, and coordination between various levels of healthcare service providers. In rural areas, township health centers and village health offices are responsible for delivering primary healthcare and public health services. In urban areas, community health service centers and stations provide these services (2, 3). Achieving “fair health protection for all” has become a critical objective in developing the healthcare system under the new healthcare reform. To attain this goal, integration of urban and rural healthcare systems is viewed as essential in achieving the convergence of the three healthcare systems (4). China’s hierarchical medical care system possesses distinct characteristics when compared to other countries. It prioritizes the provision of basic healthcare services through primary care institutions, with referrals to higher-level hospitals made when necessary. Other nations possess dissimilar models of hierarchical care that vary according to their healthcare systems, cultures, and economic situations.

The correlation between economic development and the medical system presents a multifaceted and crucial problem, comprising economic growth, societal welfare, population health, medical resource allocation in rural and urban areas, as well as medical service demand and supply. Firstly, economic development provides the essential material basis and social framework for the establishment and improvement of both rural and urban medical structures. The formation and evolution of medical systems are influenced by the economic level, economic structure, and economic system. Moreover, the construction and improvement of urban and rural medical systems significantly affect economic development. The standardization and efficiency of the hierarchical medical system, the quality and accessibility of medical facilities, and the coverage and level of medical insurance all have the potential to affect health status, consumption patterns, labor supply, human capital, and scientific and technological innovation in both rural and urban areas (5).

Since the reform and opening up, China’s economic development has exhibited regional disparities, leading to significant variations in healthcare services and economic environments among different provinces. Overall, China’s regional development policy divides the country into four major economic regions: Eastern, Middle, Western, and Northeastern based on its economy (6). This classification method was released by the Central Committee of the Communist Party of China and has been widely used in various economic statistical analyses for China, including “*Opinions on Promoting the Rise of the Central Region*” and “*Opinions on the Implementation of Certain Policies and Measures for the Development of the Western Region*” issued by the State Council of the People’s Republic of China. In terms of regional characteristics, there is a significant developmental disparity between the Eastern and Western regions of China, with a tendency toward complexity. Regional development disparities are significant, leading to unequal opportunities for regional growth. In the context of regional coordinated development, China has formulated plans to develop the western region, make new strides in promoting the revitalization of the northeast, expedite the rise of the central region, and prompt the eastern region to accelerate modernization.

China’s medical insurance system has long been characterized by a dual structure and fragmentation between urban and rural areas. The increase in medical resources and services in urban areas has far outpaced that in rural areas, and has shown a tendency to expand, causing an imbalance in medical resources between urban and rural areas (7). The disparities in fragmentation are evident in the significant variations in the treatment of individuals enrolled in the basic medical security system. The issue of being unable to access fair medical security rights and benefits is highly prominent, significantly impeding the integrated development of urban and rural areas. Therefore, examining the relationship between economic development and the effectiveness of implementing a hierarchical health care system can broaden our understanding of the functions and responsibilities of the health care system. It can help evaluate the efficiency and effectiveness of medical systems in both urban and rural areas, examine reforms and advances in the medical field, and provide evidence-based policy recommendations to facilitate coordinated development of the economy and society.

In this context, this paper aims to explore the relationship between the implementation of the hierarchical medical system and economic development from the perspective of urban and rural areas. It analyses the mechanism and degree of influence of hierarchical medical system on economic development, and the constraints and conditions promoting economic development within hierarchical medical system. The goal is to provide valuable insights for improving the hierarchical medical system, enhancing the efficiency and quality of medical services, and promoting sustainable economic development. There are two main aspects to the research significance of this work: It enriches and expands the theoretical research on the relationship between urban and rural hierarchical medical systems and economic development and provides innovative perspectives on health care evaluation and reform. Additionally, it examines crucial challenges in building China’s hierarchical healthcare network, providing valuable direction to innovate and optimize the system. It also provides policy guidance for China and other developing countries in formulating and implementing health care systems. For coordinating and integrating urban and rural health systems with economic development, it provides valuable references and lessons, as well as useful suggestions and programs.

## 2 Data, variables, and methods

### 2.1 Data sources

In 2009, the pilot of China’s hierarchic care policy was first implemented, marking the beginning of its impact. The public health data for this study were obtained from China Health Statistical Yearbook published from 2008 to 2022. The public health data used in 2022 were referenced from the 2022 Statistical Bulletin of Health in the People’s Republic of China, which was published on October 12, 2023, by China’s Department of Planning, Development, and Informatization. The 2022 China Statistical Yearbook was used to gather data on the economy. Except for the province (region)-based effect evaluation analysis, which we can only find the annual data from 2021, the remaining studies employed time-series analyses covering the period from 2009 to 2022.

### 2.2 Study design

**Figure 2:**
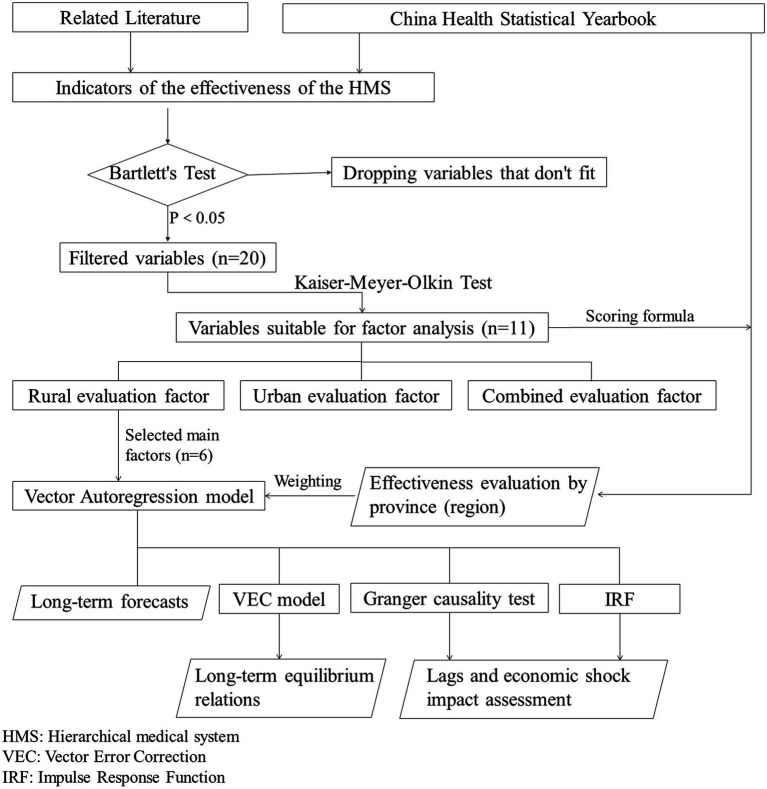
Research design flowchart.

The overall flow of this study is clearly represented in Figure 2. Since there are some variables that may not be suitable for direct time series regression analysis, the principal component analysis (PCA) was used to reduce the number of independent variables through dimensionality reduction processing. This was done to improve the generality and applicability of the model. The Kaiser-Meyer-Olkin test was conducted on the filtered variables to determine the most appropriate variables for factor analysis. The maximum value of the rotated factor matrix for each component is the main influencing factor for that component. The factor analysis method can be used to establish a model for evaluating the effectiveness of a hierarchical medical system. The scoring formula can be used to analyze the implementation effectiveness more intuitively in each province (region). Based on the extracted variables and corresponding weighting coefficients, a Vector Autoregression model can be constructed to accurately evaluate economic lags, covariance, and predict potential future scenarios.

If many variables are selected, factorial downscaling must be performed before conducting econometrics-related time series regressions. This is necessary to avoid pseudo-regression problems and enhance the quality of the analysis. Factor analysis is a statistical method used to reduce multidimensional variables to a smaller set of common factors through correlation analysis. These factors are then analyzed and processed. The main concept is to break down the original variables into two components: one component is the linear combination of the common factors, which contains the majority of the information from the original variables; the other component consists of the unique factors that are not associated with the common factors, representing the disparity between the linear combination of the common factors and the original variables. P-dimensional variables *x* = [*x*_1_, …, *x_i_*, …, *x_p_*] T are modeled by a factor analysis as follows:


$$\left[\begin{array}{c} x1\\ x2\\ xp \end{array}\right]=\left[\begin{array}{ccc} a11 & a12 & a1m\\ a21 & a22 & a2m\\ ap1 & ap2 & apm \end{array}\right]\left[\begin{array}{c} f1\\ f2\\ fp \end{array}\right]+\left[\begin{array}{c} \varepsilon 1\\ \varepsilon 2\\ \varepsilon p \end{array}\right]\;\;\;or\;\;\;x=Af+\varepsilon$$

The key to constructing a factor analysis model for the multidimensional variable *x* lies in solving the factor loading matrix A and the common factor vector *f*. The typical steps involved in factor analysis are as follows: (1) Selection of variables for analysis. Factor analysis requires a strong correlation between observed variables, which can be determined through qualitative and quantitative analysis. If there is little or no correlation between the variables, they will not share a common factor. Therefore, it is essential to have a strong correlation between the original variables. In order to eliminate the effects caused by the units of the dependent variable, it is necessary to standardize the data. This involves standardizing each variable, denoted as *x*, to have a mean of zero and a variance of one. The standardization can be expressed in the following form:


$$x_{ij}=\frac{\left(x_{ij}-\frac{1}{n}\sum_{j=1}^{n}x_{ij}\right)}{\sqrt{\frac{1}{n-1}\times \sum_{j=1}^{n}{\left(x_{ij}-\frac{1}{n}\sum_{j=1}^{n}x_{ij}\right)}^{2}}}$$

(2) Calculate the correlation coefficient matrix (S). It can help determine whether there is a correlation between the original variables, which is crucial for factor analysis. If there is no relationship between the selected variables, it would be inappropriate to conduct factor analysis. The elements can be represented in the following form:


$$S_{ij}=\frac{1}{n-1}\overset{n}{\underset{k=1}{\sum }}x_{ik}\times x_{ik}$$

(3) Extracting common factors. Through coordinate transformation, the goal is to minimize the number of factors between closely related original variables. This makes it easier to explain the actual meaning of the factor solution. The eigenvalues of the correlation coefficient matrix (S) are decomposed. The first largest eigenvalues (m) are used to estimate the factor loading matrix. *λ_j_* represents the standard deviation and the variance of each vector of the common factor is set to 1. The expression for the factor loading matrix is denoted as *A*.


$$A=\left[\sqrt{\lambda 1\times }\gamma 1\times \sqrt{\lambda 2\times }\gamma 2\times \ldots \ldots \times \sqrt{\lambda m}\times \gamma m\right]$$

(4) Calculate factor scores. The specific scores of the original variables on the common factors can be estimated by regression methods:


$$fj=A^{T}S^{-1}xj$$

According to this formula, the factor scores for each variable can be derived. These factor scores can serve as a reference for selecting variables for regression analysis. The variables extracted by the factor analysis method can already reflect most of the information, and the variables are reduced by factor down scaling to improve the accuracy of the VAR analysis. Vector Autoregression (VAR) is a model proposed by Christopher Sims in 1980 for forecasting interconnected time series systems and estimating endogenous relationships among joint variables (8). The VAR approach constructs a model by treating each endogenous variable as a lagged function of all the endogenous variables in the system, thus avoiding the need for a structured model. With the recent advancements in development, the VAR model has evolved from a two-dimensional model to a multidimensional model and has gained widespread usage in the field of economic time series analysis (9). Mathematical expression of the VAR(p) model with a lag of order p is:


$$y_{t}=\varphi_{1}y_{t-1}+\ldots +\varphi_{p}y_{t-p}+A_{p}x_{t}+\varepsilon_{t}\;\;,t=1,2,3,\ldots ,T$$

In the formula, *y_t_* is the k-dimensional endogenous variable; *x_t_* is the k-dimensional exogenous variable; *A_p_* is the matrix of coefficients to be estimated; *p* is the lag order; *ε_t_* is the vector of perturbed columns, *t* is the number of samples, and *φ* is the k × k-dimensional matrix coefficients to be estimated. The VAR model can provide a detailed reflection of the dynamic relationship between the implementation effect of a hierarchical medical system and economic development. It can also examine the existence of lagged effects of economic development, as compared to Ordinary Least Squares regression (OLS).

After constructing the VAR model, it is important to assess whether there is a long-term stable relationship among multiple variables. If a cointegrating relationship is identified, an error correction model (VEC) can be constructed. Variables with cointegration do not necessarily have a causal relationship. Therefore, it is necessary to conduct a Granger causality test to determine whether there is a statistically significant causal relationship between the variables. The Granger causality test is used to examine the statistical time sequence. However, determining a causal relationship requires consideration of authentic experience and relevant theories. The Impulse Response Function (IRF) Analysis is used to analyze the dynamic influence relationship between variables. This is done by applying a shock to the ε_t_ term in the VAR model, which allows for the depiction of current and future values of the variables. Change to reflect the trend of current and future values obtained. The prerequisite for using the Granger causality test and IRF analysis is that the VAR model must be stable.

### 2.3 Variables selection

**Figure 3:**
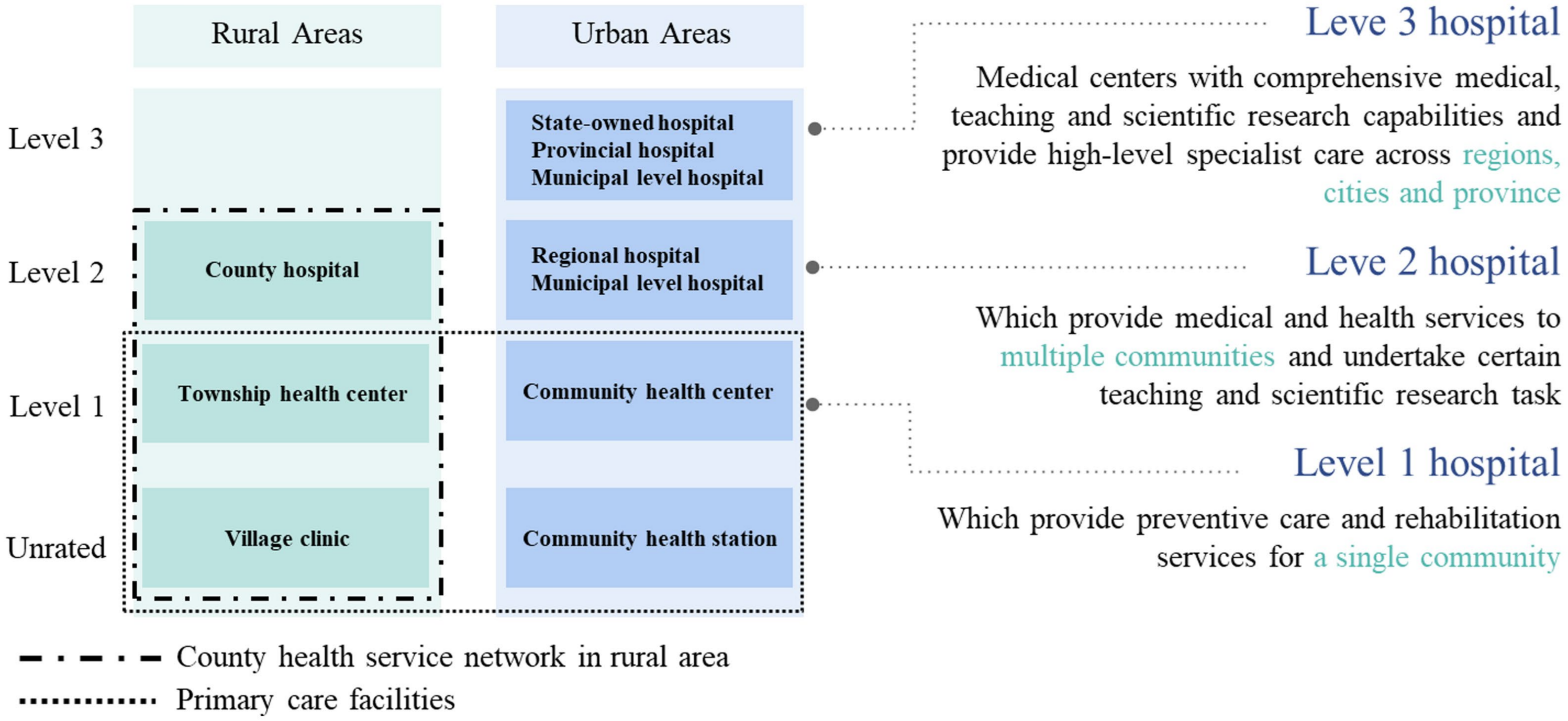
Levels and types of medical institutions in China.

According to Chapter 6 of the China Health Statistical Yearbook (2022), titled “Primary Health Care Services,” variables were selected for screening to be included in the construction of the hierarchical medical system effect model. Bartlett’s test was first performed on the selected variables to remove irrelevant variables. As shown in Figure 3, some variables were grouped into primary healthcare institutions, township health centers, community health service centers, and hospitals (10). According to Yongchuang Gao et al., severe job stress among healthcare workers, the high cost of some healthcare services, and the need for improvement in the development level and service capacity of primary hospitals are important factors influencing the effectiveness of hierarchical treatment (11). We considered selecting appropriate variables from physical resources, human resources, financial support, service utilization, and other relevant indicators (10, 12).

Currently, there is no comprehensive system available for referencing the selection of indicators to measure the effectiveness of the hierarchical medical system in China (13). At the same time, the government typically utilizes primary care in basic medical units and referral rates as performance appraisal standards for evaluating the hierarchical medical system. This has led to the formation of a mandatory hierarchical medical system due to inflexible policy constraints (3). Some regions have implemented various measures to enhance primary care in basic medical units and increase referral rates to meet performance targets, making it difficult to evaluate the true impact of the hierarchical medical system. At the same time, these two indicators are too limited and do not fully reflect the actual impact of hierarchical medical system (14).

Through a combination of literature review and statistical methods (KMO > 0.6, *p* < 0.05), the 11 chosen variables and their respective descriptive statistics are displayed in Table 1. Except for figures indicating bed utilization, average hospitalization days, and infectious disease incidence, all other indicators manifest a steady and annual upward trend. Healthcare burden is more pronounced in rural localities, registering approximately 3.5%. First, *PriPerson* and *HospPerson* are two indicators that allow us to measure the level of human resources at various levels of healthcare organizations. Based on the influencing factors of health in China and relevant references, such as the urban and rural hierarchical medical systems, The quantity of primary care physicians is an indicator of the human resources available within the primary institutions of a medical system that operates under a hierarchical model. A comparison of this quantity with the number of hospital physicians can illustrate any disparity between the two categories.

**Table 1:** Selected variable and descriptive statistics.

Index selection	Variable name	*N*	Min	Max	Mean	SD
Number of practicing (assistant) physicians in basic medical and health institutions	*PriPerson*	12	949,054	1,614,973	1198901.58	226,225
Number of practicing (assistant) physicians in hospitals	*HospPerson*	13	1,198,542	2,396,771	1737964.38	404,101
Burden of medical care on urban residents (%)	*UrbanBurden*	13	4.21	5.39	4.79	0.382
Burden of medical care on rural residents (%)	*RuralBurden*	13	5.51	8.87	7.36	1.090
Beds utilization rate for community health services (%)	*CommBeds*	14	41	60	52.25	5.793
Beds utilization rate in rural health center (%)	*RuralBeds*	14	47	63	57.69	5.219
Beds utilization rate in hospital (%)	*HospBeds*	14	71	90	82.96	6.381
Average hospitalization days for community health services	*CommDays*	12	9.5	10.4	9.93	0.270
Average hospitalization days in rural health center	*RuralDays*	14	4.8	6.6	6.09	0.569
Number of people receiving health education	*EduPerson*	9	1,115,470	2,091,000	1,475,448	326,561
Incidence rate of statutory infectious diseases (1:100,000)	*Incidence*	13	190.36	263.52	224.68	19.30

*UrbanBurden* = Expenditures on health care for urban residents/Urban consumption expenditure.

*RuralBurden* = Expenditures on health care for rural residents/Rural consumption expenditure.

Secondly, *UrbanBurden* and *RuralBurden* enable us to gage the proportion of disposable income that rural and urban residents spend on healthcare expenses. Considering China’s economic development, the medical burden is also a crucial factor that affects the functioning of the hierarchical medical system (15). This is particularly significant for the effectiveness of rural primary health institutions.

Thirdly, *CommBeds, RuralBeds*, and *HospBeds* are key factors in assessing the implementation of the hierarchical medical system. Beds utilization rate reflects the difference between patients’ tendency to visit the first clinic and the referral effect, allowing for an evaluation of the real impact of the hierarchical medical system. In addition, *CommDays* and *RuralDays* measure the extent to which healthcare resources are utilized in urban and rural areas.

Finally, *EduPerson* and *Incidence* are also important factors and the lack of understanding among residents about the hierarchical medical system is one of the reasons that affects its effectiveness (16). China’s population growth has had an impact on the number of people receiving medical education in China. However, the extent and direction of this impact have not been uniformed, and it has been constrained and moderated by other factors. On the one hand, China’s population growth has resulted in an expansion of the pool of individuals pursuing medical education, creating a foundation and demand for human resources in the field of education. On the other hand, this population growth has also strained and imbalanced the distribution of resources for medical education, presenting elevated demands and challenges for its development. The number of people receiving medical education also reflects, to a certain extent, the healthcare disparity between urban and rural areas. The analysis of the incidence of infectious diseases, combined with people’s choice of initial care, also provides some insight into the balance of pressures between the hierarchical medical system and more advanced hospitals. The incidence rate of infectious diseases can reflect the effectiveness of the first clinic, particularly in rural areas where residents often choose the township health center as their first option. The incidence rate of infectious diseases can reflect the impact of initial healthcare visits, particularly in rural areas. The fact that residents initially opt for township health centers and access better healthcare services can help reduce the incidence rate of infectious diseases to some extent (17).

For the macro evaluation of economic growth, the most widely used and accepted indicator in existing research is gross domestic product (GDP). The GDP indicators were combined with the evaluation system constructed using factor analysis to build the subsequent VAR model.

## 3 Results

### 3.1 Analysis of HMS effects

Bartlett’s spherical test is used to assess the degree of independence between variables, while the tKMO (Kaiser-Meyer-Olkin) statistic is used to measure the degree of correlation between variables. The joint KMO and Bartlett’s Test is commonly used to assess the suitability of variables for factor analysis (KMO = 0.672, *p* = 0.032). Principal component analysis (PCA) is used to extract the common factors’ variance. The initial value of 1.000 was assigned to each variable, and the resulting values represented the unrotated variance of the common factors for each factor. The extracted values of all variables were greater than 0.9, except for *PriPerson* (0.895), *HospBeds* (0.819), *CommDays* (0.745), *EduPerson* (0.744), and *Incidence* (0.876). Each variable’s extracted value exceeded the recommended standard of 0.7, indicating that the common factor extraction was performed effectively. Calculation of the variance for each metric factor should be followed by a decomposition of the total variance. The Scree Plot displays three factors in the sample with eigenvalues greater than 1.000 (6.6918, 1.9017, and 1.1769). There is a total of two inflection points, where stone falls 1–2 are extremely prominent and stone falls 3–4 are not significant. According to Table 2, it can be concluded that a total of three common factors were extracted, which accounted for 38.132, 27.662, and 23.028% of the variance, respectively, with a cumulative contribution of 88.822%. The contribution rate of the metric factors is greater than 85%, indicating that the extracted metric factors have a strong explanatory power for the impact of the hierarchic care. The weight coefficients corresponding to the three common factors are 0.43, 0.31, and 0.26. These weight coefficients will be used in the establishment of the score factor equations and VAR models. The common factors are extracted and computed to generate the component matrices of the initial factors as follows.


$$\left[\begin{array}{ccc} 0.712 & 0.580 & -0.396\\ 0.554 & -0.118 & 0.824\\ -0.432 & 0.806 & 0.405 \end{array}\right]$$

**Table 2:** Total variance decomposition.

Initial eigenvalues				Extraction sums of squared loadings			Rotation sums of squared loadings		
Factor	Total	Variance (%)	Cumulative (%)	Total	Variance (%)	Cumulative (%)	Total	Variance	Cumulative (%)
1	6.692	60.835	60.835	6.692	60.835	60.835	4.194	38.132	38.132
2	1.902	17.288	78.123	1.902	17.288	78.123	3.043	27.662	65.794
3	1.177	10.699	88.822	1.177	10.699	88.822	2.533	23.028	88.822
4	0.576	5.232	94.054						
5	0.352	3.197	97.251						
6	0.167	1.519	98.770						
7	0.064	0.582	99.352						
8	0.049	0.442	99.794						
9	0.012	0.108	99.902						
10	0.008	0.076	99.978						
11	0.002	0.022	100.00						

The extraction method is the main component method.

**Figure 4:**
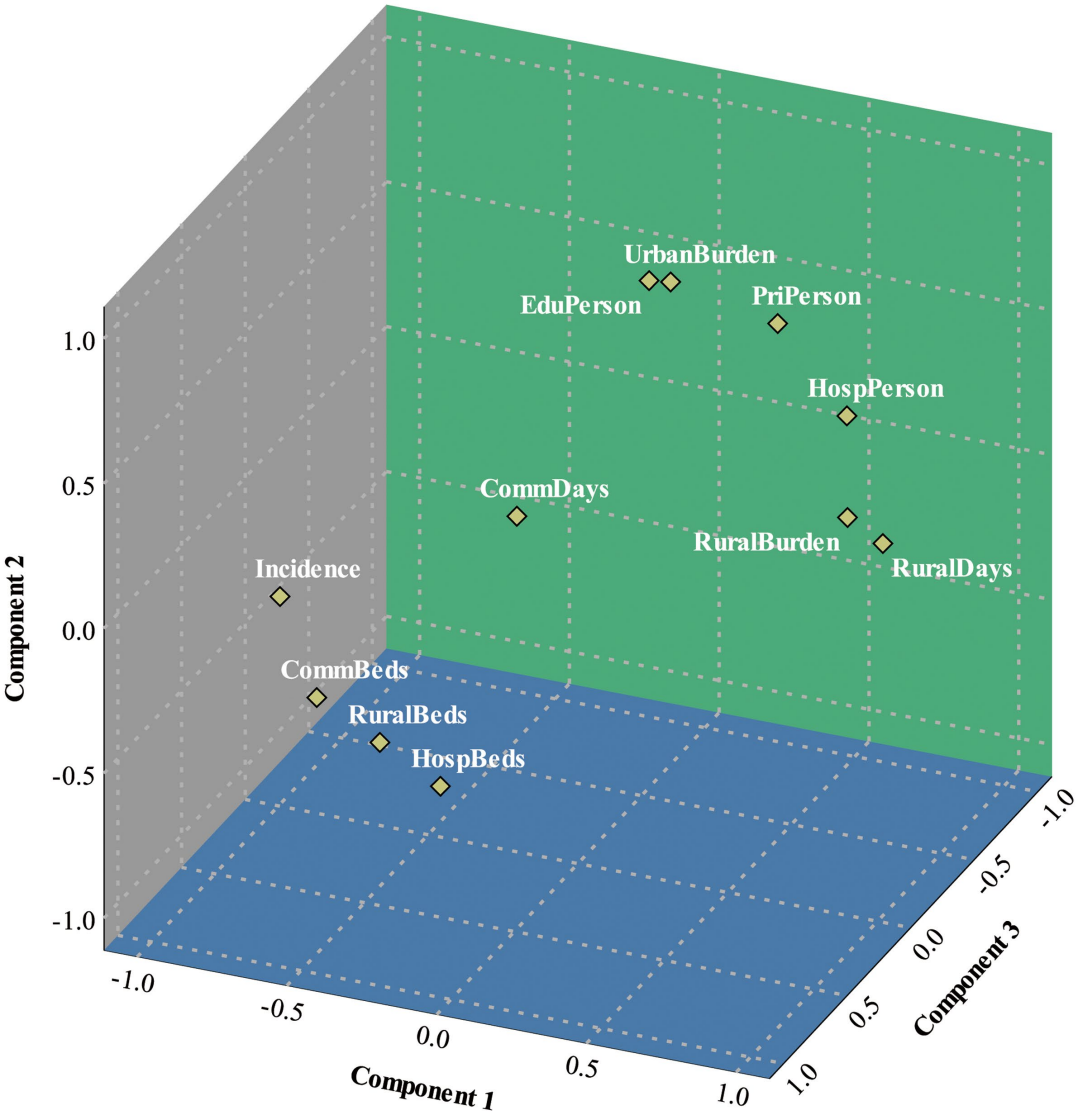
Component diagram in rotated space.

The next step involves rotating the matrix of initial factors. Depending on the size of the indicator’s share of the rotated factor loading matrix, the corresponding principal factor can be defined (18). According to the rotated factor loadings matrix (Table 3) and as depicted in the factor loadings plot (Figure 4), the first principal factor has a significant weight in the loadings of the two indicators: *RuralDays* (0.953) and *RuralBurden* (0.927). The first principal factor reflects the varying levels of development of provinces and cities in promoting a hierarchical medical system in rural areas. Therefore, it is defined as the “rural evaluation factor.” The second main factor has a significant weight of loadings in the two indexes: *UrbanBurden* (0.923) and *EduPerson* (0.819). Therefore, the second principal factor reflects the varying levels of development in promoting hierarchical diagnosis and treatment in urban areas across each province and city. As a result, it is defined as the “urban evaluation factor.” We can find that residents in urban areas are more affected by economic factors, while those in rural areas are more influenced by time considerations (19). The third main factor carries a significant weight in the two indexes of *RuralBeds* (0.849) and *CommBeds* (0.749). The utilization rate of primary care beds in both urban and rural areas can effectively reflect the efficiency of primary care. The loading weight of *HospBeds* (0.704) is second only to the above two indexes. However, the simultaneous appearance of all three indexes will increase the difficulty of analysis and potential covariance problems. The third main factor primarily evaluates the effectiveness of primary care, so this index is omitted. We defined the third principal factor as the “combined evaluation factor.”

**Table 3:** Factor matrix and rotated factor matrix.

Factor matrix				Rotated factor matrix			
	1	2	3		1	2	3
*PriPerson*	0.919	−0.038	0.222	*PriPerson*	0.537	0.717	−0.305
*HospPerson*	0.953	0.241	−0.018	*HospPerson*	0.819	0.510	−0.186
*UrbanBurden*	0.762	−0.005	0.596	*UrbanBurden*	0.282	0.923	−0.064
*RuralBurden*	0.808	0.531	−0.134	*RuralBurden*	0.927	0.298	0.064
*CommBeds*	−0.863	0.345	0.303	*CommBeds*	−0.554	−0.297	0.749
*RuralBeds*	−0.756	0.575	0.188	*RuralBeds*	−0.301	−0.354	0.849
*HospBeds*	−0.709	0.555	−0.086	*HospBeds*	−0.160	−0.546	0.704
*CommDays*	−0.265	−0.762	−0.306	*CommDays*	−0.479	−0.311	−0.647
*RuralDays*	0.804	0.389	−0.383	*RuralDays*	0.953	0.112	−0.153
*EduPerson*	0.666	−0.213	0.505	*EduPerson*	0.138	0.819	−0.234
*Incidence*	−0.853	−0.158	0.351	*Incidence*	−0.846	−0.194	0.350

Three components were extracted, and the rotation has converged after 5 iterations.

The three metrics mentioned above can comprehensively explain the impact of the hierarchical medical system in each province and city, considering both urban and rural perspectives. Considering the development of China’s hierarchical medical system, evaluating the utilization and effectiveness of primary medical service institutions can provide an accurate reflection of the current situation. China’s large agricultural population and the unequal allocation of healthcare resources across regions make it important to comprehensively analyze the situation, with a focus on both urban development and rural areas (20). In view of the nature of China’s population distribution, *per capita* indicators, utilization rates, and ratios have been prioritized over simple quantitative indicators when selecting indicators.

Based on the matrix of component score coefficients and the corresponding weight coefficients, the three principal factor scores, denoted as *F*_1_, *F*_2_ and *F*_3_, can be obtained separately as follows:


$$\begin{array}{l} F_{1}\;\left(Rural\right)=0.005\;PriPerson+0.178\;HospPerson\\ \;\;\;\;\;\;\;\;\;\;\;\;\;\;\;\;\;\;\;\;\;\;\;\;\;\;\;\;\;\;\;-0.139\;UrbanBurden+0.290\;RuralBurden\\ \;\;\;\;\;\;\;\;\;\;\;\;\;\;\;\;\;\;\;\;\;\;\;\;\;\;\;\;\;\;\;-0.102\;CommBeds+0.018\;RuralBeds\\ \;\;\;\;\;\;\;\;\;\;\;\;\;\;\;\;\;\;\;\;\;\;\;\;\;\;\;\;\;\;\;+0.118\;HospBeds-0.138\;CommDays\\ \;\;\;\;\;\;\;\;\;\;\;\;\;\;\;\;\;\;\;\;\;\;\;\;\;\;\;\;\;\;\;+0.339\;RuralDays-0.176\;EduPerson\\ \;\;\;\;\;\;\;\;\;\;\;\;\;\;\;\;\;\;\;\;\;\;\;\;\;\;\;\;\;\;\;-0.265\;Incidence \end{array}$$

$$\begin{array}{l} F_{2}\;\left(Urban\right)=0.234\;PriPerson+0.056\;HospPerson\\ \;\;\;\;\;\;\;\;\;\;\;\;\;\;\;\;\;\;\;\;\;\;\;\;\;\;\;\;\;\;\;\;+0.475\;UrbanBurden-0.055\;RuralBurden\\ \;\;\;\;\;\;\;\;\;\;\;\;\;\;\;\;\;\;\;\;\;\;\;\;\;\;\;\;\;\;\;\;+0.111\;CommBeds+0.028\;RuralBeds\\ \;\;\;\;\;\;\;\;\;\;\;\;\;\;\;\;\;\;\;\;\;\;\;\;\;\;\;\;\;\;\;\;-0.155\;HospBeds-0.186\;CommDays\\ \;\;\;\;\;\;\;\;\;\;\;\;\;\;\;\;\;\;\;\;\;\;\;\;\;\;\;\;\;\;\;\;-0.217\;RuralDays+0.417\;EduPerson\\ \;\;\;\;\;\;\;\;\;\;\;\;\;\;\;\;\;\;\;\;\;\;\;\;\;\;\;\;\;\;\;\;+0.176\;Incidence \end{array}$$

$$\begin{array}{l} F_{3}\;\left(Combined\right)=0.006\;PriPerson+0.042\;HospPerson\\ \;\;\;\;\;\;\;\;\;\;\;\;\;\;\;\;\;\;\;\;\;\;\;\;\;\;\;\;\;\;\;\;\;\;\;\;\;\;\;\;\;\;\;-0.158\;UrbanBurden+0.136\;RuralBurden\\ \;\;\;\;\;\;\;\;\;\;\;\;\;\;\;\;\;\;\;\;\;\;\;\;\;\;\;\;\;\;\;\;\;\;\;\;\;\;\;\;\;\;\;+0.305\;CommBeds+0.359\;RuralBeds\\ \;\;\;\;\;\;\;\;\;\;\;\;\;\;\;\;\;\;\;\;\;\;\;\;\;\;\;\;\;\;\;\;\;\;\;\;\;\;\;\;\;\;\;+0.253\;HospBeds-0.420\;CommDays\\ \;\;\;\;\;\;\;\;\;\;\;\;\;\;\;\;\;\;\;\;\;\;\;\;\;\;\;\;\;\;\;\;\;\;\;\;\;\;\;\;\;\;\;-0.011\;RuralDays+0.042\;EduPerson\\ \;\;\;\;\;\;\;\;\;\;\;\;\;\;\;\;\;\;\;\;\;\;\;\;\;\;\;\;\;\;\;\;\;\;\;\;\;\;\;\;\;\;\;+0.103\;Incidence \end{array}$$

The following formula for the composite score can be obtained by using the variance contribution of the common factors as weights.


$$F=\frac{F_{1}\times 0.38132+F_{2}\times 0.27662+F_{3}\times 0.23028}{0.88822}$$

From the formula, Table 4 provides the factor scores and rankings of 31 provinces (including municipal cities directly under the central government) in China. From a rural perspective, the top five provinces in terms of the effectiveness of hierarchical medical system implementation are Shandong, Guangdong, Henan, Jiangsu, and Hebei. The top five provinces in terms of the effectiveness of implementing an urban hierarchical medical system are Henan, Sichuan, Shandong, Hunan, and Guangdong. Additionally, the five provinces with the highest implementation effectiveness, based on the bed utilization rate as the principal factor, are Shandong, Guangdong, Henan, Jiangsu, and Sichuan. Hebei Province’s hierarchical medical system is well implemented in rural areas, but its effectiveness in urban areas is ranked ninth. The regions that have achieved great implementation results are mainly located in the middle and eastern parts of the country, which reflects the imbalanced allocation of healthcare resources and services in China (21, 22). On the one hand, the imbalance of the hierarchical medical system is reflected in the concentration of medical resources in urban areas. In Shanghai, for example, the average length of hospitalization is as high as 160.9 days in 2021, which is significantly higher than the national average. On the other hand, the utilization rate of hospital beds in Beijing and Shanghai, as well as in developed coastal provinces, is higher than that in underdeveloped areas (3, 23). However, the implementation of the goal of “linking primary care institutions and hospitals” is not effective in these areas. This is due to the lack of awareness among residents about the hierarchical medical system, as indicated by the second main factor. In addition, Grade A tertiary hospitals and abundant medical resources are typically concentrated on the east coast and in the affluent middle regions. As a result, residents tend to prefer these hospitals for treatment (24).

**Table 4:** Factor scores and rankings of provinces (regions).

		F_1_ (rural)	Rank	F_2_ (urban)	Rank	F_3_ (combined)	Rank	*F*	Rank
Eastern region	Beijing	20181.673	17	14529.695	22	4963.584	19	14476.024	20
	Tianjin	9270.998	27	6836.014	27	2309.844	27	6707.917	27
	Hebei	45736.024	5	38153.045	9	11338.919	6	34456.646	6
	Shanghai	15051.552	24	11442.971	26	3696.620	24	10983.844	25
	Jiangsu	49087.384	4	42118.516	6	12201.554	4	37354.033	5
	Zhejiang	41840.029	7	34356.287	11	10376.319	7	31352.069	8
	Fujian	19950.251	18	16939.846	20	4996.293	18	15135.750	19
	Shandong	61645.201	1	50028.159	3	15253.011	1	45999.640	2
	Guangdong	57405.569	2	46633.448	5	14226.442	2	42856.163	3
	Hainan	5260.454	28	4448.190	29	1384.796	28	4002.686	28
Northeastern region	Liaoning	23670.995	12	17012.312	19	5839.350	13	16974.235	17
	Jilin	15686.416	22	12745.979	24	3910.236	23	11717.577	23
	Heilongjiang	17378.331	20	12553.299	25	4293.120	21	12483.190	22
Middle region	Shanxi	20503.672	15	23907.446	17	5259.928	15	17611.621	16
	Anhui	31356.232	9	42011.757	7	8138.361	9	28655.234	9
	Jiangxi	20201.891	16	25876.913	15	5248.416	16	18092.434	14
	Henan	53974.514	3	65795.955	1	13821.014	3	47245.887	1
	Hubei	30821.750	10	41858.646	8	8023.999	10	28348.444	10
	Hunan	34959.178	8	46757.600	4	9095.378	8	31928.131	7
Western region	Inner Mongolia	15230.261	23	18752.270	18	3952.643	22	13403.291	21
	Guangxi	24078.103	11	37960.541	10	6412.201	11	23821.473	11
	Chongqing	16770.377	21	24624.497	16	4453.631	20	16023.160	18
	Sichuan	45572.000	6	63336.742	2	11864.207	5	42365.421	4
	Guizhou	19132.892	19	26290.063	14	5020.000	17	17702.954	15
	Yunan	22899.790	13	33178.582	12	6021.783	12	21725.140	12
	Tibet	1850.917	31	2476.707	31	546.750	31	1707.690	31
	Shaanxi	21918.292	14	29737.779	13	5704.083	14	20149.832	13
	Gansu	12817.043	25	16889.980	21	3363.861	25	11634.652	24
	Qinghai	3306.694	30	3950.545	30	938.641	30	2893.268	30
	Ningxia	4053.149	29	5048.941	28	1090.338	29	3595.132	29
	Xinjiang	12707.439	26	14491.223	23	3307.819	26	10826.020	26

Provinces (regions) are excluded from Hong Kong, Macau, and Taiwan.

There is an imbalance in the distribution of medical resources in China, with significant disparities in the allocation of medical institutions between urban and rural areas. Health inequalities have risen in China and are more pronounced among rural residents than urban residents (25). Currently, medical resources in China are more concentrated in urban areas, so the extent of coverage of the hierarchical medical system in rural areas at this stage can reflect the urban–rural gap (26, 27). At this stage, the concentration of more medical resources in rural areas indicates a well-balanced construction of the hierarchical medical system between urban and rural areas in that region. Based on the above information and by combining the characteristics of the scoring formula, we have designed the Urban–rural Disparity Rate (ΔD) to reflect the disparity between urban and rural hierarchical diagnosis and treatment. When ΔD = 0, it indicates no urban–rural disparity in the province’s (regions) Hierarchical medical treatment. A higher ΔD value signifies a greater urban–rural gap in the effectiveness of hierarchical medical system, with medical resources being overly concentrated in urban areas and resulting in poorer implementation of hierarchical medical treatment in rural areas. The formula for calculating the value of D is as follows:


$$\Delta D=\left(\frac{F_{2}\times 0.27662-F_{1}\times 0.38132}{F_{1}\times 0.38132+F_{2}\times 0.27662}+\frac{1}{2}\right)\times 100\%$$

**Figure 5:**
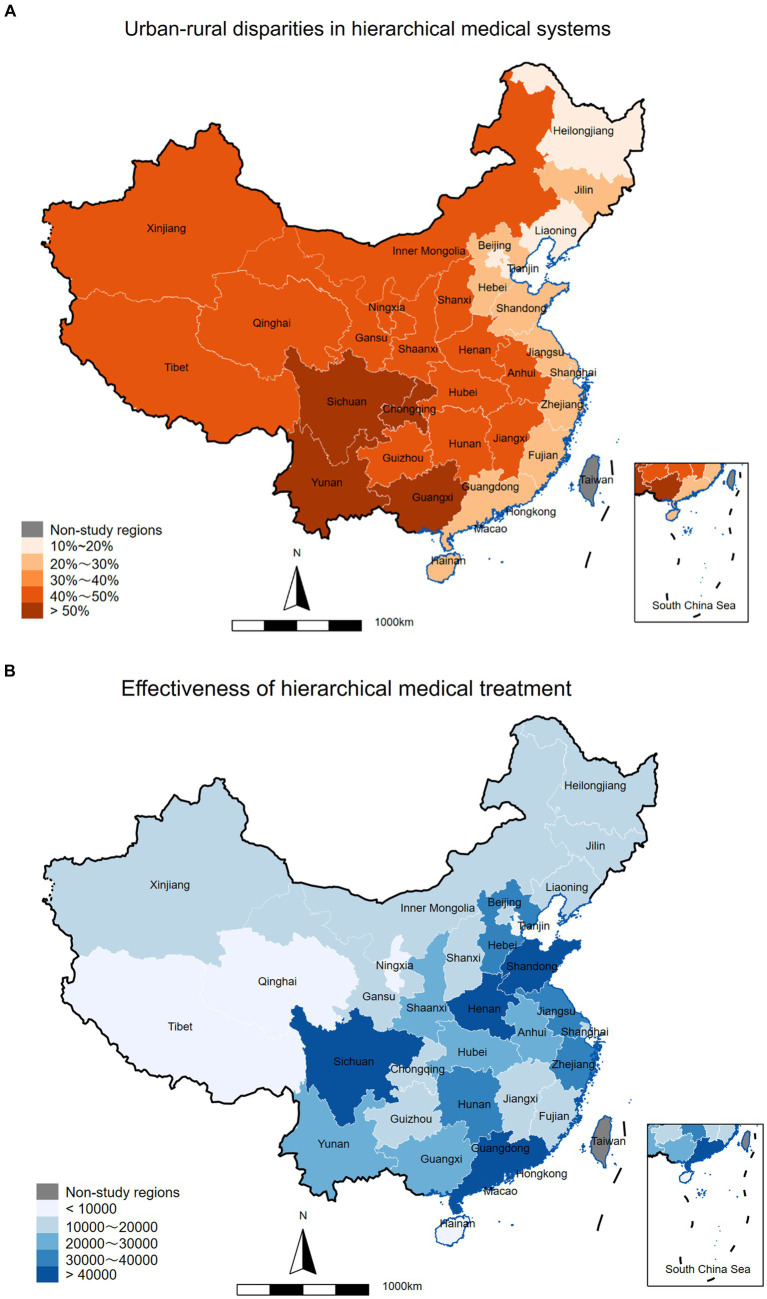
China’s HMS urban–rural gap and effect map. Urban–rural disparities in hierarchical medical system **(A)**. Effectiveness of hierarchical medical system **(B)**.

The geostatistical map of urban–rural gap from hierarchical medical system, based on the ΔD value, is presented in Figure 5A. Additionally, the graph also depicts the geostatistical map of the comprehensive efficacy of hierarchical medical system, evaluated by the *F* value (Figure 5B). The Northeast showed the lowest rural–urban disparities in HMS (ΔD_max_ = 18.77%), while the Western region had the largest disparities (ΔD_max_ = 56.70%). In comparison, the Eastern region (ΔD < 26.04%) had smaller disparities than the Middle region (ΔD < 49.25%). Upon comparison, it is evident that the ΔD value in the Eastern and Northeastern region are lower, suggesting a greater allocation of hierarchical diagnosis and treatment resources to rural areas. However, there exists a significant urban–rural disparity in the implementation of hierarchical diagnosis and treatment in the Western region of China. In Western region, the lack of widespread adoption of graded diagnosis and treatment between urban and rural areas can be attributed to the region’s geographic location, topography, pace of economic development, and policies (21). Henan (ΔD = 43.86%) and Sichuan (ΔD = 50.41%) demonstrate robust performance in the overall ranking of the effectiveness of hierarchical medical system, but the disparities between urban and rural areas are notably significant. Conversely, Shandong (ΔD = 24.11%) and Guangdong (ΔD = 24.16%), situated in the Eastern coastal region, not only secured the 2nd and 3rd positions, respectively, in terms of the efficacy of hierarchical medical system, but also directed their resources more toward rural areas. Despite the loss of labor force and an aging population in the Northeast, the hierarchical medical system does not exhibit a significant urban–rural disparity from now. The economically developed Eastern coastal regions of China possess ample health funds, enabling them to allocate sufficient resources to the establishment of rural primary healthcare institutions during the implementation of the hierarchical diagnosis and treatment policy.

According to the 2021 China Statistical Yearbook, the Eastern region has the highest GDP *per capita* (118,000 yuan), the Northeastern region has the lowest GDP *per capita* (64,000 yuan), and the Middle (59,000 yuan) and Western regions (56,000 yuan) have similar GDP *per capita*, with GDP *per capita* growth rates higher than China’s average in all economic regions. The primary objective of the Special Transfer Payments system’s design is to enhance the capacity of local governments in underdeveloped areas to deliver essential public goods to the population and to promote inter-regional equalization of public services (28). The financial expenditures of certain provinces in the Middle and Western region exhibit a high level of dependency on special transfer payments from the Eastern coastal region, as indicated by the data on special transfer payments by provinces (29). This dependency underscores the substantial disparity in economic development between the Midwest and Eastern regions. The Western region lacks the necessary funds to construct rural primary healthcare institutions, resulting in a significant disparity in healthcare development between urban and rural areas. Administration should address supply spillovers by reallocating funds or implementing fiscal policies in the affected regions, such as through the transfer of expenditures (30). When spillovers occur in the supply of healthcare in a particular region, adjacent regions will experience varying degrees of impact. Consequently, it is imperative to promptly contract or decentralize fiscal spending in line with macroeconomic market control to attain a rational and equitable distribution of expenditures across regions.

### 3.2 Benchmark regression results

We selected the principal factors *RuralBurden* and *RuralDays* (*W*_1_ = 0.42), *UrbanBurden* and *EduPerson* (*W*_2_ = 0.31), and *RuralBeds* and *CommBeds* (*W*_3_ = 0.26) to be processed by the natural logarithm of the weights. The logarithm of China’s GDP was chosen for the economic indicators. These variables are denoted as follows: *ln GDP*, *ln RuralBurden*, *ln RuralDays, ln UrbanBurden*, *ln EduPerson*, *ln RuralBeds*, *ln CommBeds*. Firstly, the ADF unit root test is performed on the processed data. *ln GDP* (*Z* = −2.866, *p* = 0.0495), *ln RuralDays* (*Z* = −5.050, *p* < 0.01), *ln EduPerson* (*Z* = −104.603, *p* < 0.01) are stable time series, and the ADF test value of *ln RuralBurden* (*Z* = −1.663, *p* = 0.4503), *ln UrbanBurden* (*Z* = −0.814, *p* = 0.8151), *ln RuralBeds* (*Z* = 0.579, *p* = 0.9871), *ln CommBeds* (*Z* = 0.137, *p* = 0.9685), are still higher than the critical value. This indicates that the logarithmic form of the variable is a non-stationary series with a unit root. After performing the first-order differencing, the variable becomes smooth at a 5% significance level. This suggests that the time series is a single integer series of the same order.

The lag order was determined by calculating the LR statistic and the Final Prediction Error (FPE), based on the Akaike Information Criterion (AIC) and Schwarz Criterion (SC) information criteria. We constructed two VAR models for testing and discovered the following: when estimating the VAR model without differencing (using a first-order lag), the AR roots of the stability test satisfied the condition that the polynomial coefficients were less than 1. This indicates that the VAR model is stable. Additionally, we conducted a Wald lag-exclusion statistics test on the joint rows of the coefficients for each equation at each order. The results showed that equation is highly significant (Prob> *χ*^2^, *p* = 0.000). The goodness of fit for the VAR equations analyzing economic growth and coefficient of determination is 99.8, 99.21, 98.37, 90.69, 94.85, 88.96, and 94.65%. These high percentages indicate a strong relationship between the variables and demonstrate that the VAR equations effectively capture the dynamics between them. When the VAR model was constructed after applying first-order differencing to all variables. However, the third-order lag exhibited a unit root, rendering the model unavailable. The first-order lagged VAR system was found to be more stable. But Wald test revealed that the features in the rural burden variable were insignificant. Therefore, for the accuracy of the analysis, we selected the undifferentiated first-order lagged VAR model for subsequent analysis and prediction.

Since there is a single integrating series of the same order, a possibility of a cointegrating relationship could exist. The Johansen cointegration test reveals a long-run cointegrating relationship between *lnGDP*, *ln RuralBurden*, *ln UrbanBurden* and *ln EduPerson* (χ^2^ = 0.058, *p* = 0.9963). When considering *lnGDP* is considered as the response variable, the vector error-correction (VEC) model can be expressed as follows (standard deviation in parentheses).


$$\begin{array}{l} lnGDP=0.4863\;\ln\;RuralBurden\;\left(3.662367\right)\\ \;\;\;\;\;\;\;\;\;\;\;\;\;\;\;\;\;\;\;\;\;\;\;+0.1187\;\ln\;UrbanBurden\;\left(3.093283\right)\\ \;\;\;\;\;\;\;\;\;\;\;\;\;\;\;\;\;\;\;\;\;\;\;-0.3492\;\ln\;EduPerson\;\left(3.631538\right)\\ \;\;\;\;\;\;\;\;\;\;\;\;\;\;\;\;\;\;\;\;\;\;\;\;-15.80497 \end{array}$$

An increase in the number of people receiving health education has a positive effect on economic growth. Meanwhile, the equation reflects the contradictory relationship between government health expenditure and economic development. For every 1% reduction in health care expenditures for urban or rural residents, GDP needs to sacrifice 0.1187% or 0.4863% of its growth rate. Additionally, the economic cost of reducing health care expenditures for rural residents is four times higher than that for urban residents.

**Figure 6:**
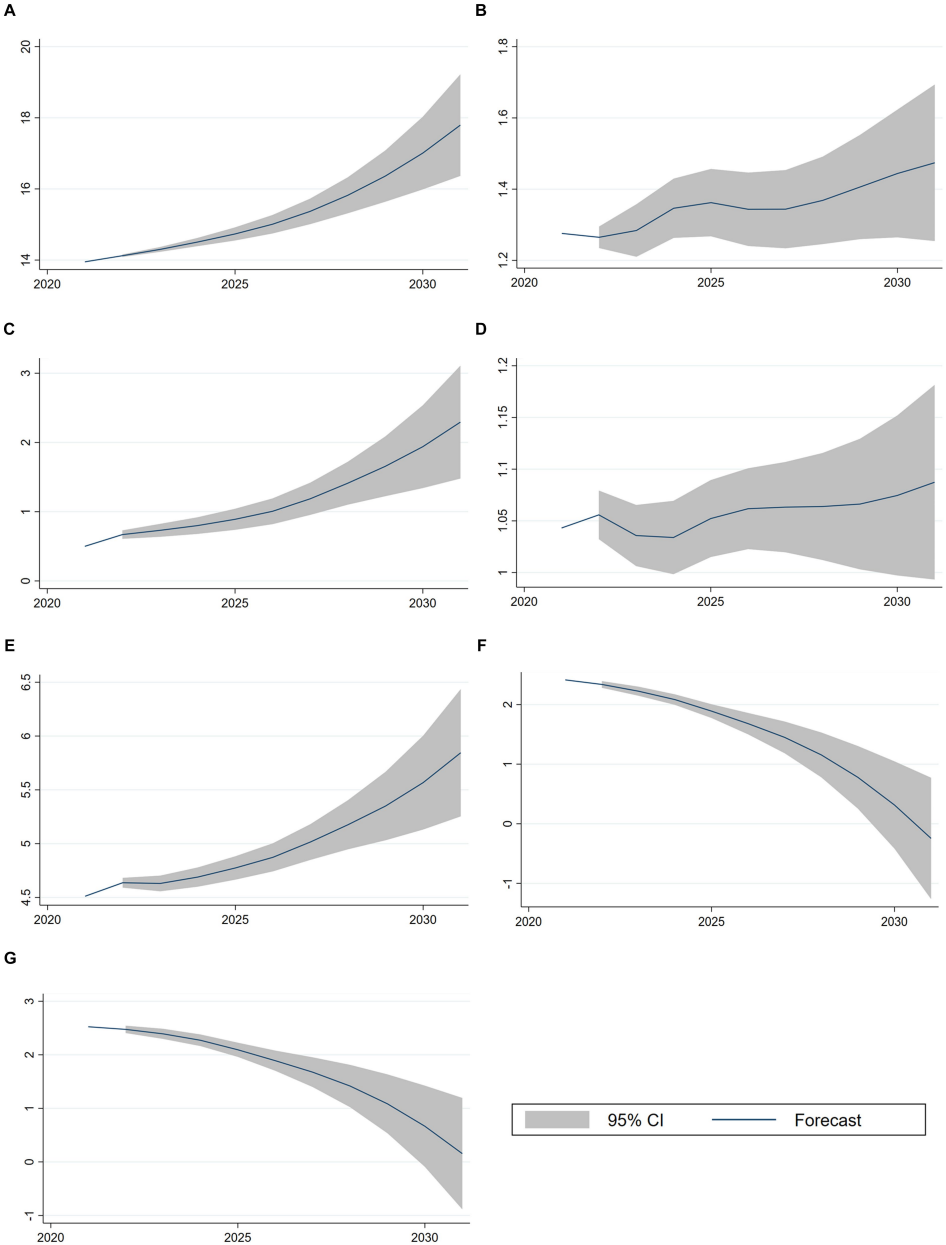
VAR regression forecast results. **(A)** Forecast for *ln GDP*. **(B)** Forecast for *ln RuralBurden*. **(C)** Forecast for *ln UrbanBurden*. **(D)** Forecast for *ln RuralDays*. **(E)** Forecast for *ln EduPerson*. **(F)** Forecast for *ln CommBeds*. **(G)** Forecast for *ln RuralBeds*.

According to the time series regression model, we can forecast the next eight periods from 2023 to 2030. As shown in Figure 6, As China’s economy is projected to continue experiencing steady growth in the future (Figure 6A), which is expected to significantly reduce the burden of medical care for rural residents (Figure 6B) compared to urban residents (Figure 6C). This indicates that the increasing medical burden on urban residents is a significant threat to the effectiveness of the hierarchical medical system. Furthermore, the gap between urban and rural medical expenditures is showing a favorable trend of decrease (31). The increase in the healthcare burden of urban residents is not significantly related to hospitalization. This is because the forecast data for average hospitalization days (Figure 6D) indicates that there will not be a significant increase in hospitalization days over the next 8 years. The number of people receiving health education (Figure 6E) is expected to increase in the future, which is a positive factor supporting the implementation of a hierarchical medical system for urban residents. Growth rate of bed utilization is decreasing in both urban (Figure 6F) and rural areas (Figure 6G), with a slightly lower rate of decline in rural areas compared to urban areas. Primary care beds in both urban and rural areas will reach saturation levels by 2030 and are growing at a decreasing rate each year. Combined with the analysis of the number of primary health care institutions, hospitals, and beds, the reason for this phenomenon is related to the implementation of the up-and-down linkage policy. This policy leads to a decrease in the bed utilization rate due to an increase in the rate of referrals. In addition, excessive medical burden is also a principal factor affecting the effectiveness of the hierarchic care policy.

### 3.3 Influencing mechanism analysis

The equation of the cointegration relationship indicates a long-term dependence between GDP and the burden of healthcare, as well as the number of urban and rural residents with medical education. To further investigate the causal relationship between these variables, the Granger causality test is required. The sensitivity of the lag order will affect the accuracy of the test results. Therefore, by selecting different lag orders for the test can obtain results with higher credibility. In this study, the lag order is 1 ~ 3 participants were selected for the test, and granger causality test results are shown in Table 5, the level of significance is 0.05. Firstly, when the lag order is 1 ~ 2. There is a mutual causal relationship between the health insurance burden of rural residents and economic development. However, the health insurance burden of urban residents is only a one-way Granger cause of economic development. It shows that in the short term, the main factor affecting economic development is the health insurance burden of both urban and rural residents. But economic development is not significantly impacted by an increase in the health insurance burden of urban residents. This means that the health insurance burden of rural residents is more influenced by economic development (32).

**Table 5:** Granger causality Wald tests.

Equation	Excluded	Chi2	lag order	Prob > χ^2^	Grande Causation
*ln GDP*	*ln RuralBurden*	13.072	1	0.000	Presence***
*ln GDP*	*ln RuralBurden*	1.6206	2	0.203	Absence
*ln RuralBurden*	*ln GDP*	59.441	1	0.000	Presence***
*ln RuralBurden*	*ln GDP*	32.939	2	0.000	Presence***
*ln RuralBurden*	*ln GDP*	0.2567	3	0.612	Absence
*ln GDP*	*ln RuralDays*	2.3747	1	0.123	Absence
*ln RuralDays*	*ln GDP*	0.6851	1	0.408	Absence
*ln GDP*	*ln UrbanBurden*	5.015	1	0.025	Presence*
*ln GDP*	*ln UrbanBurden*	1.3805	1	0.240	Absence
*ln UrbanBurden*	*ln GDP*	2.3431	1	0.126	Absence
*ln GDP*	*ln EduPerson*	2.8304	1	0.092	Absence
*ln EduPerson*	*ln GDP*	14.712	1	0.000	Presence***
*ln EduPerson*	*ln GDP*	12.098	2	0.001	Presence***
*ln EduPerson*	*ln GDP*	1.9752	3	0.160	Absence
*ln GDP*	*ln RuralBeds*	2.8431	1	0.092	Absence
*ln RuralBeds*	*ln GDP*	0.5333	1	0.465	Absence
*ln RuralBeds*	*ln GDP*	7.2265	2	0.007	Presence**
*ln RuralBeds*	*ln GDP*	70.741	3	0.000	Presence***
*ln GDP*	*ln CommBeds*	2.9419	1	0.086	Absence
*ln CommBeds*	*ln GDP*	0.0357	1	0.850	Absence
*ln CommBeds*	*ln GDP*	3.0336	2	0.082	Absence
*ln CommBeds*	*ln GDP*	1184.9	3	0.000	Presence***

* Significant at 5% level. ** Significant at 1% level. *** Significant at 0.1% level.

The presence of individuals with medical education has a unidirectional Granger effect on economic development, while there is no causal relationship between the average duration of hospitalization in rural primary health care institutions and economic development. When using the hospitalization rate of basic medical and health institutions as an indicator to evaluate the effectiveness of the hierarchic care policy, it is observed that there is no causal relationship between the hospitalization rate of rural and urban basic medical and health institutions and economic development when considering a lag order of one period. As the lag order increases, economic development becomes a unidirectional Granger cause of the hierarchical medical system’s effect, and there is a tendency for GDP growth to have a smaller lag in rural areas compared to urban areas when affecting the hierarchical medical system. To summarize, the relationship between the impact of a hierarchical medical system and economic development in rural areas is more sensitive, with a smaller time lag compared to urban areas. The short-term effects of hierarchical medical system policies on economic development are more pronounced in rural areas, while long-term and delayed effects are observed in urban areas.

**Figure 7:**
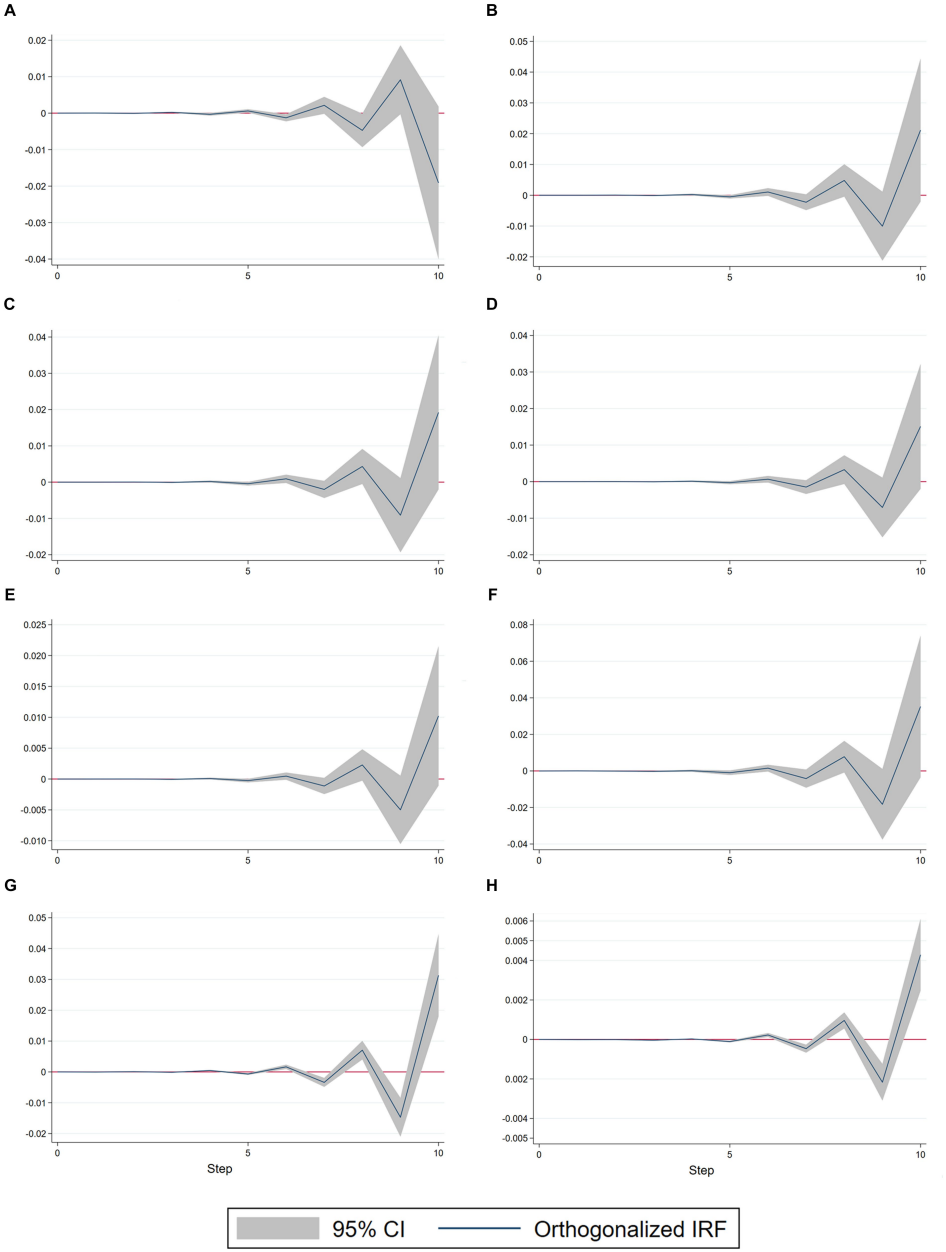
Impulse response function (IRF) results. **(A)** *ln GDP*, *ln RuralDays*. **(B)** *ln GDP*, *ln RuralBurden*. **(C)** *ln GDP*, *ln UrbanBurden*. **(D)** *ln GDP*, *ln EduPerson*. **(E)** *ln GDP*, *ln RuralBeds*. **(F)** *ln GDP*, *ln CommBeds*. **(G)** *ln RuralBurden*, *ln GDP*. **(H)** *ln UrbanBurden*, *ln GDP*.

The Impulse Response Function (IRF) Analysis reflects the path of the propagation of the effect of the perturbation term ε_t_ on each endogenous variable, which spans a number of tracking periods of 10 years. Figure 7 illustrates the relationship between GDP and the indicators of the hierarchical medical system implementation. Among these indicators, the burden of medical care for urban and rural residents has a bidirectional impact on GDP, while the other indicators have a unidirectional impact. Notably, the impact pattern of GDP on the average number of days of hospitalization in the township (Figure 7A) differs from the other indicators. Initially, there is no response in the current period, followed by oscillations and fluctuations from period 2 to 5. In period 6, there is a weak negative impact. This is followed by a positive shock in period 6, a negative fluctuation in period 7 with a response current period of −0.005, followed by a sharp positive shock with a value of 0.01, and an immediate shift to a significant negative fluctuation with a response current period of −0.02 in year 10. Thus, the overall impact of the GDP shock on the average number of days of hospitalization in townships is negative and has a long-time lag. The graphical fluctuations of the impact of GDP on the other indicators are the same (Figures 7B–F), with only differences in the intensity of the shocks. The initial response is zero in the current period. The impulse response function shows minor oscillatory fluctuations in periods 1–5. There is a negative shock in the seventh period, followed by a positive fluctuation with a larger shock effect in the eighth period. All indicators had a response of 0.01 for the current period except for *ln Ruralbeds* (0.0025). All observations experience a negative shock in the ninth period, and then a positive shock with a large value in the tenth period. The effect of the shocks is significant for all indicators, except for the overall weak impact of GDP shock (−0.01 to 0.020) on the rural hospitalization rate (Figure 7E). Finally, the overall effects of ln GDP on *ln RuralBurden*, *ln UrbanBurden*, *ln EduPerson*, *ln RuralBeds,* and *ln CommBeds* are all positive shocks. The impact of the shocks of *ln GDP* on *ln RuralBurden* (Figure 7B) and *ln UrbanBurden* (Figure 7C) is the same.

Meanwhile, the graphical patterns of the impacts of shocks to GDP for *ln RuralBurden* (Figure 7G) and *ln UrbanBurden* (Figure 7H) are consistent with the graphical patterns of the shocks to *ln GDP* for *ln RuralBurden* and *ln UrbanBurden*. However, it is important to note that the logarithmic indicator of rural burdens is 7.5 times larger than that of the indicator of urban burdens for *ln GDP*. This result is in line with the cointegration equation and the covariance equation. The results also reflect the accuracy of the cointegration equation and the Granger causality test, as well as the stability of the VAR model construction. At the same time, also experiences small shocks with a fluctuation amplitude of less than −0.004 ~ −0.002 for other indicators, and all of them indicate negative shocks. Apart from *ln GDP* (−0.001), the negative values of all other indicators in the 10th period are less than −0.0001. To summarize, rural areas are more sensitive to changes in economic policy compared to urban areas, and the impact of shocks is more pronounced. Reducing the burden of health insurance for rural residents generates greater economic benefits than reducing the burden of health insurance for urban residents. The impulse responses between all indicators have a lag period of approximately 5 years. This means that there is a significant delay between the hierarchical medical system for urban and rural residents and economic development. Additionally, most of these responses exhibit oscillatory fluctuations with multiplier effects. Eventually, the fluctuation value keeps amplifying, producing a significant positive or negative fluctuation. Therefore, the evaluation of the effectiveness of China’s urban and rural hierarchical medical systems should not rely on short-term results alone. Instead, it should consider the long-term impacts to avoid misjudgments caused by impulsive fluctuations and lags that may convey misleading information.

## 4 Discussion

In this study, we investigated the dynamic relationship between the effectiveness of hierarchical diagnosis and treatment and economic growth from the perspective of urban and rural areas in China. We utilized a variety of statistical and econometric methods and obtained valuable findings and insights. First, we developed a model to assess the efficacy of tiered diagnosis and treatment using principal component analysis. This model integrates several factors that impact the effectiveness of tiered diagnosis and treatment and evaluates them at both urban and rural levels. This approach overcomes the limitations of previous studies that relied on a single indicator or level, enhancing the objectivity and comprehensiveness of the evaluation. At the same time, we also developed and introduced a new indicator, the Urban–rural disparity rate (ΔD), to assess the disparity between urban and rural tiered diagnosis and treatment effects in various provinces (regions). This indicator reflects the fairness and balance of the tiered diagnosis and treatment system, offering a new perspective for future research. Finally, we primarily utilized an econometric model (VAR) to examine the dynamic relationship between the efficacy of the tiered diagnosis and treatment and economic growth. We also investigated economic factors such as long-run equilibrium, short-run cointegration, causality, and response to shocks between the two, revealing their interactions and influencing mechanisms.

The main findings of this study are consistent with or complementary to the results of several other studies of the same type, indicating the reliability and validity of this study. To ensure the reliability of the research data, we have chosen to use data from authoritative Chinese government departments such as the National Bureau of Statistics and the China Health and Wellness Commission, instead of relying on statistical yearbooks from commercial organizations. There have been numerous studies demonstrating a decreasing urban–rural gap in the resources of China’s healthcare system (33, 34). At the same time, this study has some shortcomings and limitations, including the timeliness and completeness of the data, the assumptions and applicability of the model, and the selection and definition of the variables. For example, this study exclusively utilized 2021 data from each province (region) to assess the urban–rural gap in the hierarchical medical system. These aspects need further refinement and improvement in future studies. Next, we will discuss the analytical results of this paper, focusing on regional differences in the hierarchical medical system and supply and demand.

### 4.1 Regional disparities in HMS

The current unbalanced and inadequate development of health resources in China is illustrated in Figure 5. In relation to geographical distribution, Beijing Municipality and Shanghai Municipality stand out as the most densely populated and well-equipped regions in terms of health resources in China, with Beijing holding a distinct advantage. Conversely, regions such as Guangdong, Zhejiang, and Henan exhibit relatively lower levels of resource allocation (35). The observed outcome contrasts with the anticipated overall impact of hierarchical diagnosis and treatment. This discrepancy may stem from inadequate medical resources in the relevant regions, resulting in a more favorable effect of hierarchical diagnosis and treatment due to increased medical workload and challenges in accessing higher-level medical services. Studies have indicated that patients’ age, level of education, and mobility within the population have varying impacts on their willingness of hierarchical diagnosis and treatment. As patients age, they are more inclined to select a primary healthcare organization with lower healthcare utilization, as their primary healthcare demand is the management of long-term, well-defined chronic diseases. The higher the level of education of a patient, the better their understanding of their own condition, leading to a tendency to select a primary care organization that can alleviate their medical burden. Conversely, heightened population mobility within a specific area is associated with decreased awareness of local primary healthcare facilities. Instead, individuals tend to be more acquainted with community hospitals and their diagnostic and treatment capabilities, leading to a preference for primary healthcare institutions that are more convenient and familiar. Since the onset of reform and opening up in China, large cities in the nation have been experiencing problems related to limited medical resources. These resource limitations are due to rapid population growth and urban expansion (36). In Beijing and Shanghai, where Grade A tertiary hospitals are more concentrated and the economy is more developed, residents show a greater inclination toward these high-level medical institutions. The aforementioned factors can be summarized as the impact of medical service burden and the severity of the patient’s illness on the selection of the initial medical institution (16). The advancement of an economy is expected to alleviate the burden of medical services and enhance patients’ ability to assess the severity of their own illnesses. This phenomenon is observable in practice, as evidenced by the likelihood that enhancing economic development in areas with the highest concentration of healthcare resources will have a more significant impact than improving patients’ ability to judge the severity of their own illnesses by reducing the burden of medical services. This, in turn, leads to a diminished effectiveness of hierarchical diagnosis and treatment. Conversely, in regions with limited health resources, this change is expected to enhance the efficacy of hierarchical diagnosis and treatment. There have already been studies suggesting it is essential to establish medical associations and expedite the construction of multi-practice facilities (37, 38). By establishing a connected medical association, we can promote the sharing of resources between primary and high-level medical institutions, leading to a more rational allocation of resources (39).

By establishing a close medical association, it can facilitate the sharing of resources between primary medical institutions and high-level medical institutions and achieve the rational allocation of resources (40). The establishment of “medical associations” and the promotion of urban residents’ awareness of primary medical care are important strategies for enhancing the effectiveness of hierarchical medical systems (38, 41). A well-established hierarchical medical system not only alleviates the burden on high-level medical institutions but also reduces overall public health expenditures, contributing to the improvement of both the economy and the well-being of the population.

### 4.2 Supply and demand in HMS

There are numerous factors that affect the effectiveness of hierarchical diagnosis and treatment in China, which can be analyzed from both the supply and demand perspectives. Factors on the supply side include the quantity, quality, distribution, and flow of medical resources, while factors on the demand side include patients’ health status, medical behavior, preferences, and satisfaction. Based on the results of the factor analysis in Table 4, High-level medical institutions have a strong demand for medical treatment. The healthcare demand of low-level medical institutions is small, and they are medium- and short-distance medical travel. The types of medical services at different levels are mainly “low supply—low demand” and “high supply—low demand” types (42).

From a supply perspective, current increases in health care spending do not contribute to economic growth (5). Increased investment in public health has contributed to a decrease in the average health-care burden in urban and rural areas (43). This aligns with the collective understanding that narrowing the gap in disposable income contributes to economic development. By increasing tax revenues and public health expenditures, the economic development imbalance caused by the healthcare burden problem can be effectively reduced. The promotion of the hierarchical diagnosis and treatment also helps to reduce the disparity between healthcare accessibility and healthcare burden, thereby contributing to economic development. This conclusion can also be drawn from the results of the VEC model that the economic expenditure required to reduce the healthcare burden for rural residents is four times that of urban residents. The Granger causality Wald tests in Table 5 illustrate that the significance and time lag of the economic impact of increasing the burden of health insurance for rural residents is much shorter. Based on this, we can conclude that balancing the imbalance caused by economic development contributes to the effectiveness of hierarchical treatment. By enhancing the establishment of a hierarchical treatment system in rural areas, it can effectively reduce the average healthcare burden of rural residents and improve the accessibility of healthcare. This, in turn, can help to better balance the current issue of uneven economic development, achieve a reduction in the disposable income gap, and contribute to GDP growth.

From a demand perspective, patients’ medical behavior and satisfaction are crucial factors that affect the effectiveness of hierarchical diagnosis and treatment (37). If patients have low preference for and satisfaction with the hierarchical diagnosis and treatment system, it will lead to insufficient trust and support for the system (44). As a result, they will be unwilling to participate in and cooperate with the implementation of the hierarchical diagnosis and treatment system and will tend to maintain their original habits and patterns of medical care. This will result in difficulties in the promotion and popularization of the hierarchical diagnosis and treatment system. In November 2023, a cluster of respiratory disease cases among children in northern China led to extensive overcrowding and long queues at the outpatient clinics of the Beijing Children’s Hospital (45). In our assessment of the effectiveness of hierarchical medical systems in various regions, we have already discovered that residents in urban areas are more likely to opt for hospitals for medical treatment. If the hierarchical diagnosis and treatment system can be further improved in urban areas, hospital emergency departments will not be overcrowded, thereby preventing the wastage of medical resources. Therefore, it is essential to enhance health education and patient management, improve their health awareness and behavior, and guide them to seek medical treatment reasonably and adhere to the system and norms of hierarchical diagnosis and treatment.

### 4.3 Recommendations for intervention

The hierarchical medical system (HMS) in China plays a crucial role in promoting equitable healthcare and supporting economic development. Based on the findings of the study, we suggest the following possible interventions.

First, the relevant authorities should strengthen the construction of primary health care centers (PHCs). PHCs serve as the initial point of contact for healthcare services in both urban and rural areas. PHCs serve as the first point of contact for healthcare services in both urban and rural areas. Enhancing capacity, infrastructure, and workforce can improve overall healthcare delivery. Public health departments should consider allocating additional resources to PHCs, including funding, medical personnel, and equipment. Encourage community engagement and health education programs to promote preventive care. Our study highlights the disparities in the effectiveness of hierarchical diagnosis and treatment among different regions of China (Eastern, Middle, Western, and Northeastern). To address this issue, allocating more resources (e.g., medical facilities, specialists, and funding) to regions with larger disparities is a direct measure to improve interregional disparities. The reallocation of resources should be accompanied by enhanced training of healthcare professionals in areas with insufficient hierarchical treatment to improve their skills and knowledge. Information technology can also be utilized to reduce urban–rural disparities. For example, telemedicine can provide remote expert consultations to benefit rural residents.

In addition, healthcare financing reforms, health information systems, and data sharing are also important interventions to improve the effectiveness of hierarchical care. For healthcare-related companies, exploring innovative financing models to ensure sustainable healthcare financing is crucial for the sustainability of tiered care in underdeveloped regions. For governments and public health departments, financial incentives can be tied to tiered care quality indicators to enhance regional motivation for developing tiered care. At the same time, establishing a robust healthcare information system and promoting interoperability among electronic health records (EHRs) used in hospitals, clinics, and primary healthcare centers enables smooth data sharing across various levels of care. This facilitates continuity of care and informed decision-making.

Finally, promoting preventive care, health literacy, research, and innovation are also important ways to improve the quality of healthcare in China. Educating the public about preventive measures, healthy lifestyles, and early detection of diseases can improve the health of the population and enable the rational use of resources for tiered care. Investing in research to evaluate the impact of specific interventions in health management systems can optimize the process of tiered care. Innovative approaches such as community health workers, mobile clinics, and home-based care can be explored to address issues that arise during the implementation of tiered care. Successful interventions require collaboration among policymakers, healthcare providers, and communities. By implementing these recommendations, China can further enhance the effectiveness of its tiered healthcare system and promote sustainable economic growth.

### 4.4 Limitations and future research directions

However, there are some limitations to our study that should be considered. First, the quality and availability of data can significantly influence research outcomes. In our study, panel data collected from Chinese government authorities were used. However, data gaps, inaccuracies, or inconsistencies may exist. The study covers the period from 2009 to 2022, and the COVID-19 pandemic may have influenced the results. Our model does not account for the bias in the results caused by the COVID-19 pandemic. Meanwhile, China’s hierarchical medical system is dominated by health policies, and the release of new health reform policies may have future implications. Second, the current study did not consider the impact of geospatial factors in its design. For instance, the distance to primary care facilities, the accessibility of rural transportation, topography, and geomorphology all impact the implementation of hierarchical diagnosis and treatment. In future studies, we will integrate the development of spatial time series models to explore the relationship between hierarchical diagnosis and treatment and economic development. Third, China is on the brink of entering a rapidly aging society. The rising prevalence of diseases among the rising prevalence of diseases among middle-aged and older adult individuals will exert growing pressure on the medical insurance system and healthcare services. The outcomes of the economic model fail to fully consider the influence of demographic changes. In China, urban–rural disparities vary across provinces (regions) and are not uniform. Therefore, the customs and habits of different ethnic groups should be analyzed in the context of the local situation, rather than relying too much on overall conclusions drawn from macro-analysis. Finally, our study focuses on China’s urban and rural perspectives, but the findings may not be directly applicable to other countries or regions. While our research provides insights specific to China, caution should be exercised when extrapolating these findings to different contexts. Consider discussing the unique aspects of China’s healthcare system that may limit generalizability.

We should also be concerned about several potential issues. Our study identifies a long-term cointegration relationship between economic development and healthcare burden. Establishing causality, however, is challenging. Given that there may be alternative explanations and potential reverse causal links. In addition, some unmeasured variables (e.g., cultural factors, patient preferences) may affect the implementation of hierarchical treatment effects. Development indicators used to measure China’s economy include GDP *per capita*, GDP growth rate, and more. Due to the uncoordinated nature of China’s economic development, the use of GDP as an analytical indicator can only indicate the overall impact. Factors affecting GDP also include employment, prices, balance of payments, adjustment of the structure of different industries, and more. The share of GDP accounted for by the healthcare sector in different countries varies. The significance of the healthcare sector differs from one country to another. Due to the extensive workload involved in conducting this analysis, this study does not utilize multiple economic indicators to construct various VAR models. At the same time, there are variations in *per capita* consumption across different provinces or regions. Statistical analysis, such as constructing a fixed effect or random effect model, can be conducted in the next step of the study using *per capita* GDP indicators, factor scores, and other province-specific factors.

In conclusion, recognizing these limitations enhances the robustness of our research. Future studies could address these challenges by refining methodologies, expanding data sources, and considering context-specific factors.

## 5 Conclusion

According to the findings of this study, we can conclude the following. First, there is a significant disparity in the implementation of hierarchical medical system in rural and urban areas. In general, the implementation of the hierarchical medical system in rural areas is more effective than in urban areas. The main factor influencing the adoption of hierarchical medical system in rural areas is the financial burden of medical insurance expenses. Meanwhile, urban residents tend to choose higher-level hospitals rather than community health centers. In both rural and urban areas, there is a low level of social acceptance of the effectiveness of primary care institutions, and rural residents are more cost-conscious about medical expenses compared to urban residents. These two reasons have contributed to the failure of the hierarchical medical system to achieve its goal of connecting primary medical institutions with higher-level hospitals to alleviate the pressure on higher-level medical institutions.

Second, there is a mismatch of resources in the hierarchical medical system. Urban rural disparities in Northeast and Eastern region demonstrating significantly better performance than the Middle and Western region. The urban rural disparity in the hierarchical medical system is associated with the level of economic development of the region. In some major cities in China, the uneven and inadequate development has led to an imbalanced geographical distribution of Grade A tertiary hospitals. As a result, the coverage of high-grade hospitals is greater than that of primary care hospitals in certain areas. In such instances, selecting a primary care provider not only carries the potential for misdiagnosis but also does not lead to a significant reduction in cost and time spent. This exacerbates the regional imbalance in the development of healthcare resources. In this regard, policies have been implemented to accelerate the formation of healthcare consortia and multi-practice clinics.

Finally, the impact of the hierarchical medical system policy on rural residents has a greater sensitivity to the economy and a shorter lag effect. Additionally, the economy has a more pronounced effect on the medical burden of rural residents. Instead of allocating more public healthcare funds to grade A tertiary hospital, decision-making authorities should increase investment in rural primary healthcare institutions. This approach will not only have positive effects on the economy but also promote the development of a hierarchical medical system and the establishment of universal healthcare. While the economic impact of establishing hierarchical medical systems may not be immediately apparent, it is important to recognize that the overall design of China’s hierarchical medical system is rational and should be further developed. To bridge the disparity between urban and rural healthcare levels, the Chinese government should boost public health expenditures for rural residents. This will ensure that more people can access healthcare services they need in a timely and efficient manner.

## Data availability statement

Publicly available datasets were analyzed in this study. This data can be found here: 1. China Statistical Yearbook (2022), https://cnki.istiz.org.cn/CSYDMirror/Trade/yearbook/single/N2022110021?z=Z004 , 2. China Health Statistical Yearbook (2022), http://www.nhc.gov.cn/mohwsbwstjxxzx/tjtjnj/202305/6ef68aac6bd14c1eb9375e01a0faa1fb/files/b05b3d958fc546d98261d165cea4adba.pdf, 3. Statistical Bulletin of Health in the People’s Republic of China (2022), http://www.nhc.gov.cn/guihuaxxs/s3585u/202309/6707c48f2a2b420fbfb739c393fcca92/files/8a3994e41d944f589d914c589a702592.pdf.

## Author contributions

YZ: Conceptualization, Data curation, Formal analysis, Investigation, Methodology, Software, Supervision, Validation, Writing – original draft, Writing – review & editing, Project administration. QQ: Conceptualization, Formal analysis, Software, Validation, Writing – review & editing, Writing – original draft. XX: Writing – review & editing, Conceptualization, Data curation, Formal analysis. YB: Funding acquisition, Project administration, Supervision, Writing – review & editing.
